# Lhx2 Expression Promotes Self-Renewal of a Distinct Multipotential Hematopoietic Progenitor Cell in Embryonic Stem Cell-Derived Embryoid Bodies

**DOI:** 10.1371/journal.pone.0002025

**Published:** 2008-04-23

**Authors:** Lina Dahl, Karin Richter, Anna-Carin Hägglund, Leif Carlsson

**Affiliations:** Umeå Center for Molecular Medicine, Umeå University, Umeå, Sweden; University of Washington, United States of America

## Abstract

The molecular mechanisms regulating the expansion of the hematopoietic system including hematopoietic stem cells (HSCs) in the fetal liver during embryonic development are largely unknown. The LIM-homeobox gene *Lhx2* is a candidate regulator of fetal hematopoiesis since it is expressed in the fetal liver and *Lhx2^−/−^* mice die in utero due to severe anemia. Moreover, expression of Lhx2 in embryonic stem (ES) cell-derived embryoid bodies (EBs) can lead to the generation of HSC-like cell lines. To further define the role of this transcription factor in hematopoietic regulation, we generated ES cell lines that enabled tet-inducible expression of Lhx2. Using this approach we observed that Lhx2 expression synergises with specific signalling pathways, resulting in increased frequency of colony forming cells in developing EB cells. The increase in growth factor-responsive progenitor cells directly correlates to the efficiency in generating HSC-like cell lines, suggesting that Lhx2 expression induce self-renewal of a distinct multipotential hematopoietic progenitor cell in EBs. Signalling via the c-kit tyrosine kinase receptor and the gp130 signal transducer by IL-6 is necessary and sufficient for the Lhx2 induced self-renewal. While inducing self-renewal of multipotential progenitor cells, expression of Lhx2 inhibited proliferation of primitive erythroid precursor cells and interfered with early ES cell commitment, indicating striking lineage specificity of this effect.

## Introduction

The mammalian hematopoietic system continuously generate large numbers of functional erythroid, myeloid and lymphoid cells throughout life. These functional cells originate from a small number of hematopoietic stem cells (HSCs) that are maintained by a process referred to as self-renewal [1]–[3]. The molecular basis for the differentiation and self-renewal processes is largely unknown. However, the hematopoietic system including the HSCs undergoes a rapid and strictly controlled expansion during embryonic development [4]–[7], suggesting that molecular and cellular analyses of the embryonic hematopoietic system would offer insights into these processes.

The first signs of embryonic hematopoiesis is the formation of the blood islands on the yolk sac at embryonic day 7.5 (E7.5) which almost exclusively contain primitive erythroid (EryP) cells [8], [9]. EryP cells are nucleated producing the embryonic form of hemoglobin and this stage of embryonic hematopoiesis is referred to as primitive hematopoiesis. The close association between hematopoietic cells and vascular endothelium in the blood islands led Sabin almost a century ago to postulate a common progenitor cell for these cell types referred to as the hemangioblast [10], and a progenitor cell with hemangioblast properties has recently been identified [11], [12]. Intraembryonic hematopoiesis is established approximately at E10.5, initially in the so-called aorta-gonad-mesonephros (AGM) region and shortly thereafter the fetal liver becomes colonised by progenitor cells [7], [13]–[19], marking the switch from primitive to definitive hematopoiesis as the formation of the whole spectrum of hematopoietic lineages including the definitive erythroid (EryD) lineage commences at this stage. The latter cell type differs from the EryP lineage in that they are smaller, lack nucleus and produce adult hemoglobin [8], [9].

Functional analyses of growth factor receptor-ligand interactions have been informative in the understanding of cellular interactions and signalling molecules important for both early hematopoietic development and regulation of stem and progenitor cells. The receptor tyrosine kinase c-kit and its ligand Stem Cell Factor (SCF, also c-kit ligand, Steel factor or mast cell growth factor) are essential for hematopoietic development since mice lacking functional c-kit (*White Spotting* or *W* mutants) or SCF (*Steel* or *Sl* mutants) die of a severe anemia *in utero* [14], [20]. The SCF/c-kit interaction plays a critical role in the expansion of definitive hematopoietic cells in the fetal liver [21], [22], and the SCF/c-kit signalling pathway appears to be important for both self-renewal of HSCs *in vivo* [23]–[26], and differentiation of HSCs and progenitor cells *in vitro* and *in vivo* [14], [25], [27]–[30]. Another signal transduction pathway important for the development of the hematopoietic system is mediated by the glycoprotein 130 (gp130), the common receptor and signal transducer for the interleukin-6 (IL-6) family of cytokines. [31], [32]. Although the precise role of gp130 in hematopoietic stem/progenitor cells is not fully understood signalling via gp130 is important in the expansion of fetal definitive hematopoiesis [33], [34], and appears to influence their function both *in vivo* and *in vitro* [35]–[38]. Thus, signalling from the c-kit receptor and the gp130 signal transducer play an import role in the development of the hematopoietic system as well as for stem and progenitor cell function, but molecular mechanisms modulating the activities of, and the interplay between these receptors in stem cells remains to be elucidated.

Analyses of early mouse development is hampered by the inaccessibility of the early embryo and the limited amount of tissues at these stages. These aspects can be circumvented by using the embryonic stem (ES) cell system since ES cells can be differentiated *in vitro* into cystic structures called embryoid bodies (EBs), and this process mimic the essential features of the gastrulation process [39]. Similar to the embryo, the first hematopoietic precursor cell population to develop in EBs is a transient wave of the EryP lineage followed by the development of progenitor cells of the various definitive hematopoietic lineages (EryD and myeloid) [40]. Moreover, a progenitor cell population showing hemangioblast characteristics that appears prior to the development of primitive and definitive hematopoietic progenitor cell populations, has been identified in the ES system as well as in the embryo [11], [12]. Thus, the ES/EB system represents a reliable and reproducible model system for analysing the development and regulation of the early embryonic hematopoietic system.

We have previously shown that expression of the LIM-homeobox gene *Lhx2* (MGI:96785, NM_010710) in hematopoietic progenitor cells derived from ES cells differentiated *in vitro* can promote self-renewal of a rare SCF-responsive progenitor cells present in EBs [41]. These progenitor cells could be identified in clonal assays and used to establish SCF-dependent multipotential definitive hematopoietic progenitor cell (HPC) lines sharing many basic properties with normal early fetal HSCs [41]–[43]. However, these experiments were carried out by retroviral delivery of the Lhx2 cDNA to the cells and a putative contribution to the observed phenotype by inactivation of tumour suppressor genes or activation of oncogenes caused by retroviral integration has not been excluded. Moreover, expression from retroviral vectors in ES cells differentiated *in vitro* is very inefficient [41], [44], and therefore it is not possible by using the retroviral system to systematically analyse the effects of Lhx2 expression in the ES/EB system. Thus, expression of Lhx2 can generate HSC-like cell lines but the specificity and efficiency of this event is not known.

**Figure 1:**
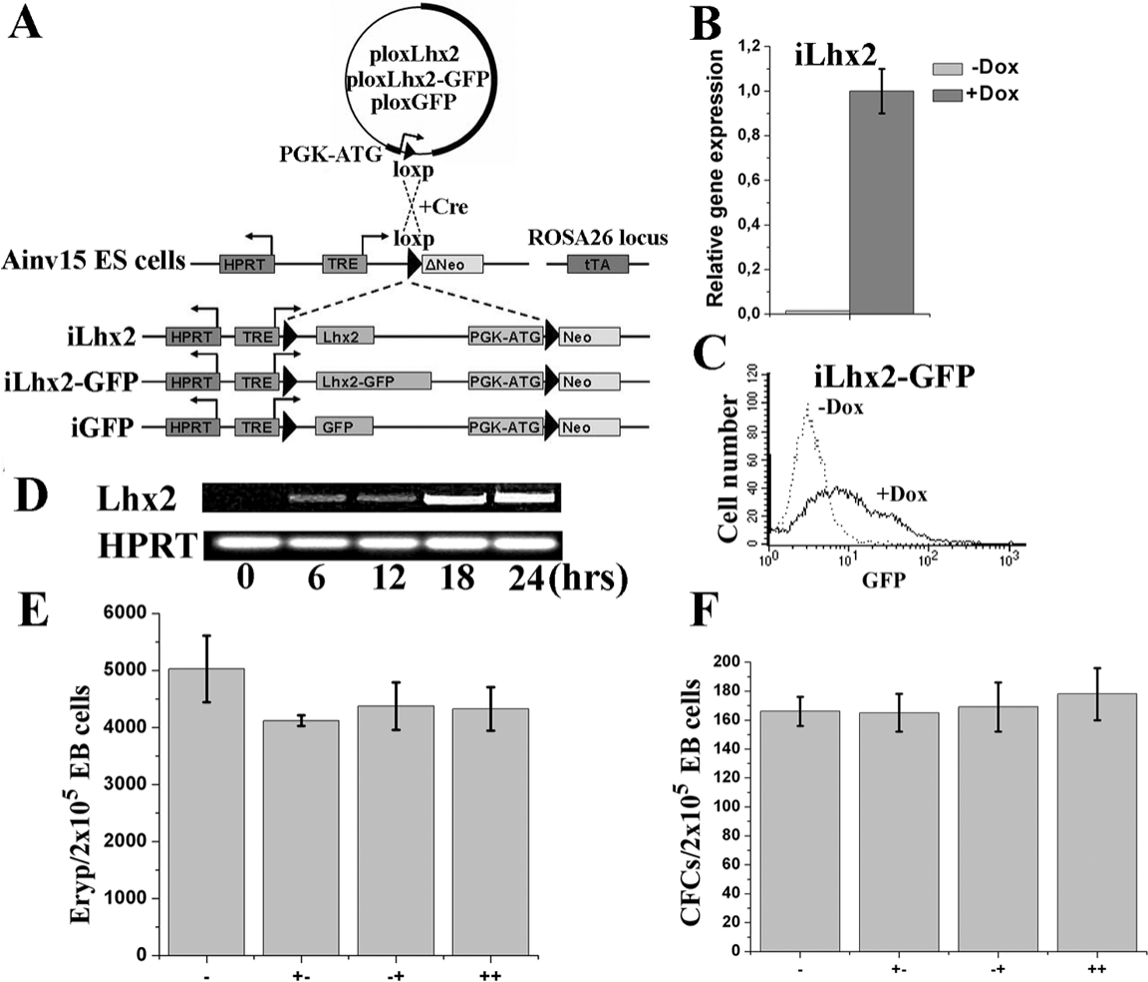
Generation of ES cell lines with inducible gene expression. A. The iLhx2, iLhx2-GFP and iGFP ES cell lines were generated by co-transfecting Ainv15 ES cells with the plox vector containing the respective gene construct together with a vector containing the Cre recombinase as described in ref. 45. The plox vector will be inserted into the loxp site down stream of the tet-responsive element (TRE) adjacent to the *Hypoxanthine-phosphoribosyltransferase* (*HPRT*) gene. The reverse tet transactivator (rTA) is inserted into the ubiquitously expressed *ROSA26* locus in the Ainv15 ES cells. B. Lhx2 expression is measured by real-time PCR analysis of iLhx2 ES cells cultured in the absence (−Dox) or presence (+Dox) of dox for 2 days. C. FACS analysis of the iLhx2-GFP ES cells cultured in the absence (-Dox) or presence (+Dox) of dox for two days. D. Lhx2 expression measured by RT-PCR at different time points (in hrs) after dox addition to iLhx2 ES cells. Expression of HPRT was used as an internal control. E. Analysis of EryP precursor cells in day 6 EB when dox is added to the control iGFP ES cells at different stages during *in vitro* differentiation. F. Analysis of definitive CFCs in day 6 EBs when dox is added to iGFP cells at different stages of ES cell differentiation. – control, no addition of dox, +− addition of dox during ES cell differentiation from day 0 to day 6, −+ addition of dox to the clonal assays of the EB cells, ++ addition of dox to both the ES cell differentiation from day 0 to day 6 and to the clonal assays of the EB cells.

To further address the role of Lhx2 in during embryonic development of the hematopoietic system, we have introduced Lhx2 cDNA into Ainv15 ES cells where its expression is efficiently regulated by a Tet-on system (Fig. 1A) [45]. Lhx2 expression directly induce self-renewal of a distinct multipotent progenitor cell within EBs in synergy with specific signalling pathways. However, Lhx2 appears to have different and even opposite effects in other cell types as its expression completely blocks proliferation of EryP precursor cells and to some extent also EryD precursor cells, and interferes with the initial steps of ES cell differentiation.

## Results

### Generation of ES cell lines with inducible Lhx2 expression

A systematic analysis of Lhx2 function in ES cells differentiated *in vitro* using retroviral vectors is not possible due to the inefficient and unpredictable expression pattern from such vectors in the ES/EB system [41]. Introduction of Lhx2 cDNA into the Ainv15 ES cells would allow for a more thorough analyses of Lhx2 function since it is possible to conditionally regulate Lhx2 expression in ES cells and during ES cell differentiation *in vitro*. Three different ES cell line were generated and referred to as iLhx2, iLhx2-GFP and iGFP ES cell lines (Fig. 1A). Efficient upregulation of mRNA and protein expression from the different cDNA constructs introduced into the respective ES cell line was achieved by adding doxycyclin (dox) to the culture media (Fig. 1B, C). Maximum Lhx2 expression was obtained within 24 hrs after dox addition and significant Lhx2 expression could be detected already 6 hrs after dox addition (Fig. 1D). Addition of dox to control ES cells (and hence GFP expression) did not interfere with any stage of ES cell differentiation *in vitro*, neither on hematopoietic commitment during EB formation nor on EryP precursor cells and definitive CFCs in clonal assays of EB cells (Fig. 1E and F). Thus, this ES cell system is amendable for systematic analysis of the specific effect(s) of Lhx2 expression at different time points during differentiation *in vitro*.

### Lhx2 expression in clonal assays of EB cells inhibit proliferation of EryP precursors and increase the frequency of definitive CFCs

**Figure 2:**
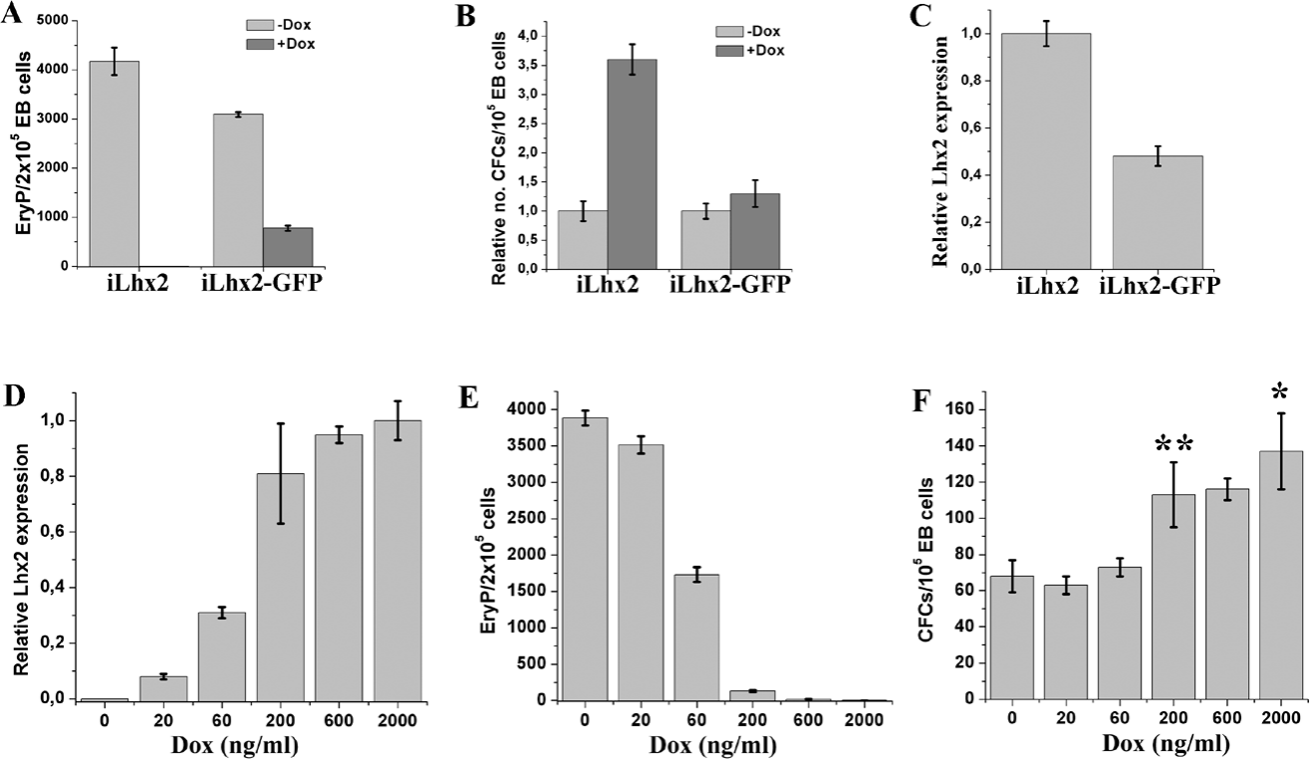
Lhx2 expression in clonal assays inhibits proliferation of EryP precursor cells and increase the frequency of definitive CFCs. A. Frequency of EryP precursor cells in day 6 EB cells generated from the iLhx2 or iLhx2-GFP ES cell lines if clonal assays were performed in the absence (−Dox) or presence (+Dox) of dox B. Relative frequency of definitive CFCs in day 6 EB cells generated from the iLhx2 or iLhx2-GFP ES cell lines if the clonal assays were performed in the absence (−Dox) or presence (+Dox) of dox. C. Relative expression of Lhx2 analysed by real-time PCR comparing iLhx2 and iLhx2-GFP ES cells cultured in the presence of dox for 2 days. D. Relative Lhx2 expression analysed by real-time PCR in iLhx2 ES cells cultured for 2 days in the presence of dox at the indicated concentrations. Maximal Lhx2 expression is reproducibly achieved in the presence of 2 µg/ml of dox which is arbitrarily set as 1. E. Frequency of EryP precursor cells in clonal assays of day 6 EBs performed in the presence of dox at the indicated concentrations. F. Frequency of definitive CFCs in clonal assays of day 6 EBs performed in the presence of dox at the indicated concentrations. *p<0,01 compared to no dox addition (0 ng/ml). **p<0,02 compared to no dox addition.

Since significant expression of Lhx2 occurred almost immediately after dox addition (within 6 hrs, Fig. 1D), we initially wanted to analyse the effect of Lhx2 expression directly on various hematopoietic progenitor cell populations by adding dox to the clonal assays performed on day 6 EB cells. The most obvious effects of turning Lhx2 expression on in clonal assays of EB cells generated from the iLhx2 ES cell lines, were a complete block in proliferation of EryP precursor cells and an approximate 3-fold increase in the frequency of definitive CFCs (Fig. 2A, B). Adding dox to EB cells generated from the iLhx2-GFP ES cell line only partially blocked proliferation of EryP precursor cells and did not significantly increase the frequency of definitive CFCs (Fig. 2A, B). Gene expression analysis showed that the iLhx2-GFP ES cells expressed approximately 50% less Lhx2 compared to the iLhx2 ES cells in the presence of saturating concentration of dox (Fig. 2C). To confirm that the difference in phenotype between iLhx2 and iLhx2-GFP ES cells was solely due to different levels of Lhx2 expression, we replated EB cells generated from the iLhx2 ES cells in clonal assays with different concentrations of dox. The level of Lhx2 expression increased with increasing concentrations of dox (Fig. 2D), and increased expression of Lhx2 expression correlated to the level of inhibition of proliferation of EryP precursor cells (Fig. 2E), and to the synergistic effect on the frequency of definitive CFCs (Fig. 2F). These results show that Lhx2 have different effects on hematopoietic precursors within EBs depending on what cells it is expressed in, and the level of expression is important for these effects.

### SCF and IL-6 is necessary and sufficient for the synergistic effect on colony formation induced by Lhx2 expression in clonal assays

**Figure 3:**
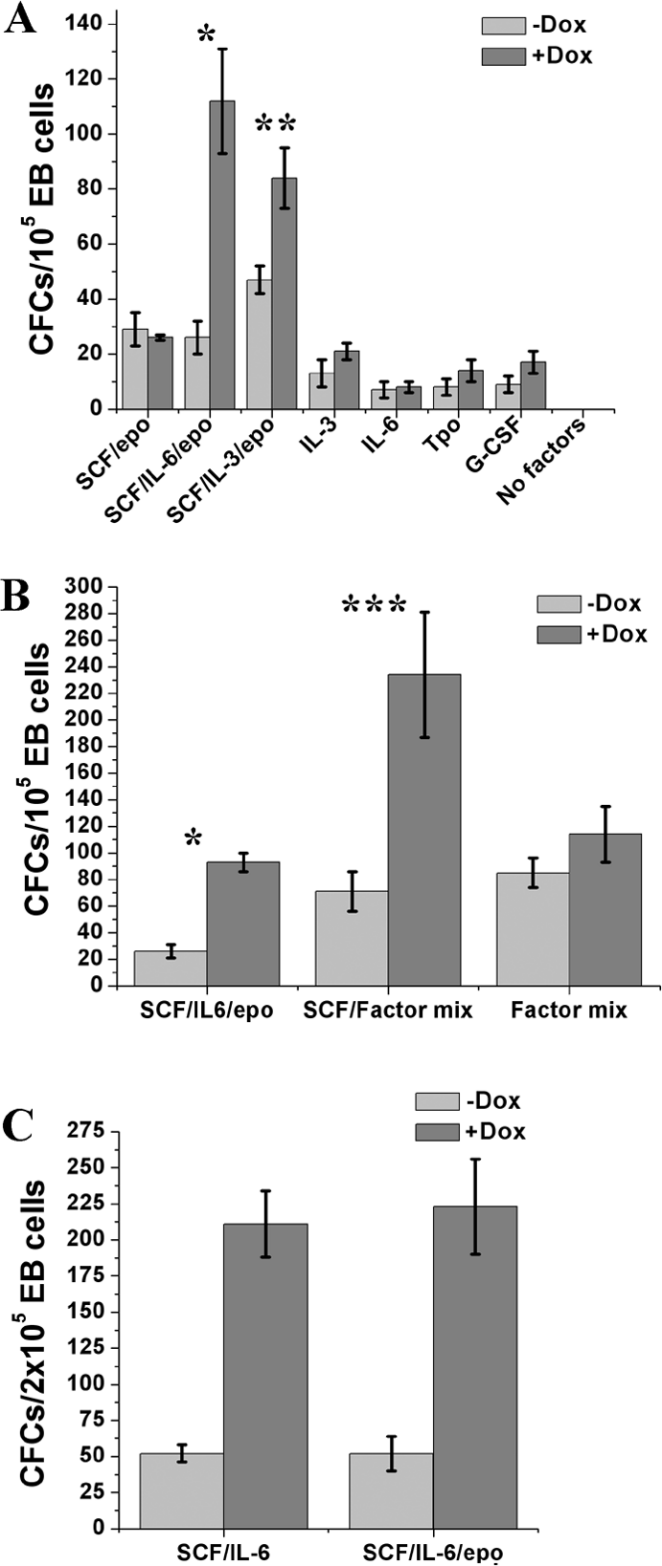
The synergistic effect of Lhx2 expression on colony formation is growth factor-specific. A. Frequency of definitive CFCs in day 6 EB cells generated from iLhx2 ES cells responding to the indicated growth factors/growth factor combinations in clonal assays performed in the absence (−Dox) or presence (+Dox) of dox. B. Frequency of definitive hematopoietic progenitor cells in day 6 EB cells responding to the indicated growth factor combinations in clonal assays in the absence (−Dox) or presence (+Dox) of dox. Factor mix is Tpo, IL-3, IL-6, Flt3L, epo. C. Number of definitive hematopoietic progenitor cells responding to the indicated growth factor combinations in the absence (−Dox) or presence (+Dox) of dox. * p<0,002, ** p<0,01, *** p<0,005.

We have previously shown that SCF is essential for the generation and maintenance of HSC-like cell lines when Lhx2 is expressed in hematopoietic cells [41], [43], [46]. We therefore wanted to elucidate whether the synergistic effect was growth factor-specific. The synergistic effect of Lhx2 expression on colony formation was observed in most factor combinations containing SCF whereas the effect was less pronounced or absent if SCF was not included in the factor combination (Fig. 3A, B). No synergistic effect of Lhx2 expression on colony formation was observed in clonal assays when EB cells were replated in SCF and epo (Fig. 3A), suggesting that activation of c-kit signalling is necessary but not sufficient for the synergistic effect of Lhx2 expression on colony formation. The least complex factor combination that consistently showed a pronounced synergistic effect on colony formation when Lhx2 expression was turned on was SCF/IL-6 (Fig. 3C). Thus, simultaneous activation of c-kit and the gp130 signal transducer is necessary and sufficient for the synergistic effect on colony formation exerted by Lhx2 expression. The definitive progenitor cells normally responding to SCF/IL-6/epo generate macrophage colonies and definitive erythroid colonies usually containing megakaryocytes and/or macrophages. Mature definitive erythrocytes assessed as hemoglobinised (red) cells rarely formed in the colonies generated in the presence of dox (data not shown), suggesting that Lhx2 expression also inhibited proliferation of the EryD precursors as was also observed for the EryP precursors cells (Fig. 2A), whereas it appears not to interfere with differentiation into mast cells, megakaryocytes, macrophages and neutrophilic granulocytes [41]. Proliferation of hematopoietic progenitor cells expressing Lhx2 in clonal assays is completely dependent on addition of growth factors since Lhx2 expression by itself did not promote proliferation (Fig. 3A, No factors). Thus, Lhx2 expression synergistically increase the frequency of CFCs in EBs and the combination of SCF/IL-6 is necessary and sufficient for this Lhx2-induced effect.

### The increase in CFCs induced by Lhx2 expression directly correlates to the efficiency in generating HSC-like cell lines

**Figure 4:**
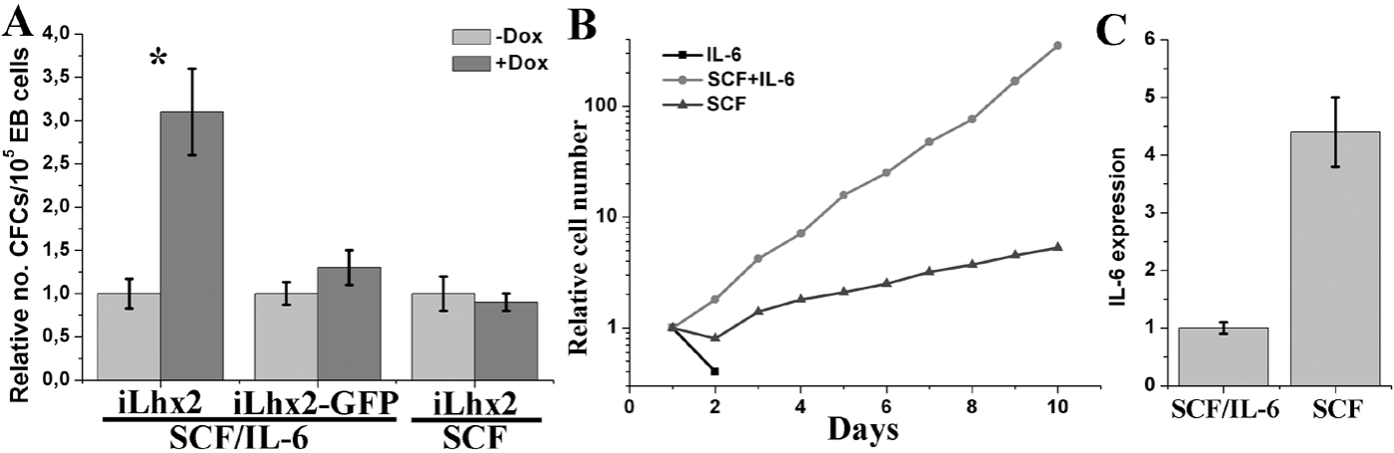
The efficiency in generating DoxHPC lines directly correlates to the synergistic effect of Lhx2 expression on colony formation, and the generation and maintenance of the DoxHPC lines is optimal in SCF/IL-6. A. Summary of the synergistic effect of Lhx2 expression (+Dox) on definitive hematopoietic colony formation of EB cells generated from the iLhx2 ES cells (high level of Lhx2 expression in 2 µg/ml of dox) or iLhx2-GFP ES cells (intermediate level of Lhx2 expression in 2 µg/ml of dox) replated in SCF/IL-6, or EB cells generated from the iLhx2 ES cells replated in SCF alone. * p<0,0001. B. Relative growth of a DoxHPC lines cultured in either IL-6, SCF/IL-6, or SCF. C. Relative expression of IL-6 analysed by real-time PCR comparing a DoxHPC line cultured either in SCF/IL-6 or in SCF for 8 days.

We have previously shown that Lhx2 expression in hematopoietic progenitor cells derived from ES cells differentiated *in vitro* and from adult bone marrow could lead to the generation of HSC-like cell lines [41]–[43], [46]. However, since we used retroviral vectors in these experiments we have not been able to exclude the contribution of secondary genetic effects caused by the fortuitous insertion of the retroviral vector into the genome leading to inactivation of tumour suppressor genes or activation of oncogenes. By using the Ainv15 ES cell lines containing the different Lhx2 constructs we could elucidate if Lhx2 expression is necessary and sufficient for generating HSC-like cell lines and the efficiency of this event. To address these issues we analysed the efficiency of establishing HSC-like cell lines from individual colonies randomly picked from the clonal assays of EBs generated from the iLhx2 and iLhx2-GFP ES cell lines (e.g. addition of dox leads to high and intermediate levels of Lhx2 expression, respectively). The generated HSC-like cell lines were denoted dox-dependent hematopoietic progenitor cell (DoxHPC) lines (see Materials and Methods and ref. [47]). The most efficient way of generating DoxHPC lines (60–69% of the picked colonies) was to transfer colonies from the clonal assays containing SCF/IL-6/epo/dox to liquid cultures containing SCF/IL-6/dox, performed on EBs generated from the ES cells expressing the highest level of Lhx2 (iLhx2), (Table 1). A low frequency (3% of picked colonies) of DoxHPC lines was obtained when the same approach was applied to the EBs generated from the ES cell line expressing intermediate levels of Lhx2 (iLhx2-GFP) (Table 1). No DoxHPC lines could be generated when colonies from clonal assays containing SCF/epo/dox of EBs generated from either iLhx2 or iLhx2-GFP ES cell line, were transferred to liquid cultures supplemented with SCF/dox (Table 1). The relative increase in CFCs when dox is added to the clonal assays containing SCF/IL-6/epo performed on the EB cells generated from the iLhx2 ES cells is on average 3,1-fold ±0,5 (9 independent experiments) as compared to when dox is not added to the clonal assays (Fig. 4A). With a 3,1-fold increase in colony formation, the increase corresponds to 68% of the total number of colony forming cells. This figure is similar to the efficiency in generating DoxHPC lines from individual colonies (60–69%) in equivalent experiments (Table 1), whereas the efficiency in generating DoxHPC lines from individual colonies was low or non-existing where limited or no synergistic effect was observed after dox addition to the clonal assays (compare Figure 4A to Table 1). Thus, the efficiency in generating DoxHPC lines directly correlated to the increase in CFCs in EBs. The frequency of CFCs in day 6 EBs when dox was added to the clonal assay was on average 8,0×10^−4^±1,3×10^−4^, hence the frequency of the Lhx2-responsive progenitor cells would be 5,44×10^−4^ (e.g. 68% of the total CFCs) or 1 in 1838 EB cells.

**Table 1:** Efficiency in generating DoxHPC lines from individual colonies in clonal assays of EB cells

ES cell line	SCF/IL-6/epo (Dox added to clonal assays)	SCF/epo (Dox added to clonal assays)	SCF/IL-6/epo (Dox added to EBs at day 4 and to clonal assay)
iLhx2	69% (66/96)*	0% (0/30)	65% (13/20)
	60% (12/20)		
iLhx2-GFP	3% (1/30)	0% (0/30)	ND

* (Number of DoxHPC lines generated/Number of colonies picked)

ND, Not done.

To analyse if the growth requirements of established DoxHPC lines were stable during culture we cultured them under different conditions. If DoxHPC lines were cultured in IL-6 alone viability rapidly decrease within 24 hrs and no live cells can be recovered after 48 hrs (Fig. 4B). If the DoxHPC lines are cultured in SCF alone the viability slightly decrease but the cells recover after a few days and eventually start to proliferate albeit at a 3–4 times slower rate compared to cells cultured in SCF and IL-6 (Fig. 4B). The recovery of the cells correlated to an upregulation of expression of the endogenous *IL-6* gene (Fig. 4C), suggesting that endogenous IL-6 expression can partly compensate for withdrawal of exogenously added IL-6. Moreover, all DoxHPC lines tested thus far rapidly differentiated into various hematopoietic lineages (usually neutrophilic granulocytes, macrophages, megakaryocytes and mast cells) upon dox withdrawal [47], suggesting that Lhx2 expression induced self-renewal of a distinct SCF/IL-6 responsive multipotential progenitor cell population present in EBs and maintenance of this progenitor cell as DoxHPC lines requires continuous c-kit and gp130 signalling.

### Analyses of the effects of Lhx2 expression at different stages of ES cell differentiation

**Figure 5:**
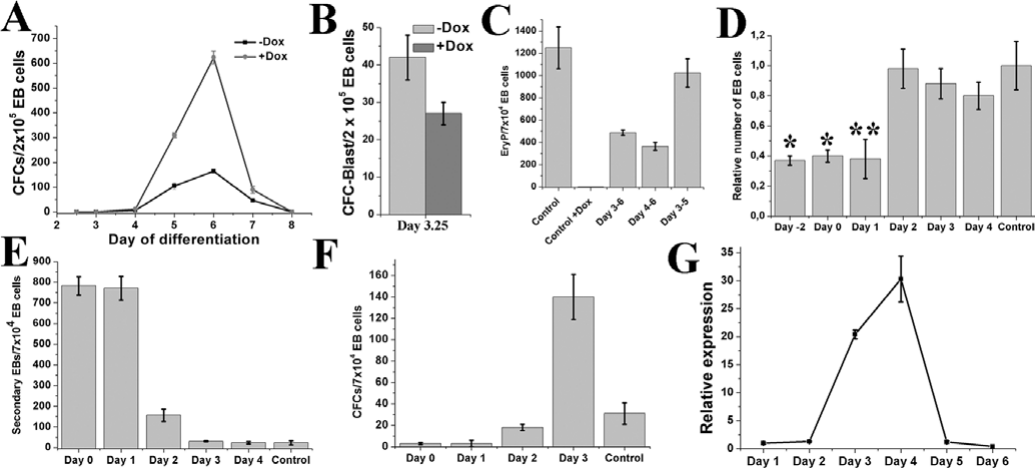
Analyses of the effect of Lhx2 expression at different stages of ES cell differentiation. A. Frequency of definitive CFCs in EB cells generated from the iLhx2 ES cells at the indicated days of differentiation starting at day 2,5 analysed in clonal assays in the absence (−Dox) or presence (+Dox) of dox. B. Frequency of CFC-Blast in day 3,25 EBs analysed in clonal assays with SCF and VEGF in the absence (−Dox) or presence (+Dox) of dox. C. Frequency of EryP precursor cells in day 6 EBs if dox is added during the indicated time points of ES cell differentiation and omitted in the clonal assays, compared to when no dox is added (Control) or if dox is added to clonal assays of control EBs (Control +Dox). The latter two control experiments are equivalent to the experiments shown in Figure 2A using the iLhx2 ES cells. D. Relative number EB cells recovered at day 6 of differentiation if dox is added to the iLhx2 ES cell line at the indicated time points during differentiation compared to when EBs are generated in the absence of dox (Control) which is arbitrarily set as 1. * p<0,005 compared to control. ** p<0,01 compared to control. E. Frequency of formation of secondary EBs in clonal assays of day 6 EBs if dox is added at the indicated time points during differentiation. Control EBs are generated without dox. F. Frequency of definitive CFCs in day 6 EBs when dox is added at indicated days of differentiation. Control EBs are generated without dox. G. Relative expression of the *Brachyury* gene during differentiation of the iLhx2 ES cell line revealing the progression of the gastrulation process in this particular ES cell line during differentiated *in vitro*.

Since Lhx2 expression appeared to promote proliferation of a specific multipotential definitive hematopoietic progenitor cell population in the EB we wanted to analyse the development of this progenitor cell during EB formation, and whether Lhx2 expression also could affect pre-hematopoietic mesoderm and/or the hemangioblast population present in the EBs approximately at day 3–4 of differentiation [11]. To start to address these issues we performed clonal assays in the presence or absence of dox on EBs generated from the iLhx2 ES cell line from day 2,5 to day 8 of differentiation. These experiments revealed that the synergistic effect of Lhx2 expression on colony formation was limited to the time points when significant numbers of definitive hematopoietic progenitor cells were detected within EBs starting at day 5 of differentiation (Fig. 5A). The most pronounced synergistic effect on colony formation of Lhx2 expression was obtained at day 6 of differentiation when the highest frequency of progenitor cells normally responding this factor combination was present in EBs (Fig. 5A). After this time point the frequency of this progenitor cell rapidly declined to be undetectable at day 8 of differentiation (Fig. 5A). To analyse the effect of Lhx2 expression on the hemangioblast population that is present during early EB development, we replated day 3.25 EB cells in clonal assays supplemented with SCF and VEGF, a factor combination that promotes proliferation of blast cell colony-forming cell (CFC-Blast) leading to formation of the blast cell colony that contains cells with hemangioblast characteristics [11]. Replating of day 3,25 EBs in this factor combination in the absence or presence of dox showed that Lhx2 expression did not have a synergistic effect, but might instead have a slight negative effect of the CFC-blast population (Figure 5B). To elucidate whether Lhx2 expression affects committed EryP precursors and/or commitment to this lineage from the hemangioblast we added dox to day 3 and 4 EBs, when commitment to this lineage occur, and replated the day 6 EBs without dox when commitment to this lineage has normally occurred. These experiments showed that significant but reduced numbers of EryP precursors are present in day 6 EBs compared to control EBs (Fig. 5C). The reduced number of EryP precursors might be due to residual Lhx2 expression in the clonal assay after dox withdrawal since this precursor appear to be sensitive to Lhx2 expression and it takes at least 24 hrs to decrease expression by 90–95% [47]. To address this issue we added dox at day 3 and withdrew it at day 5 to allow the expression level to decrease significantly before the clonal assay was performed at day 6. In this experiment the frequency of EryP precursor cells in day 6 EBs was almost equal to that of control EBs (Fig. 5C), suggesting that Lhx2 expression blocks proliferation of precursor cells committed to the EryP lineage but does not interfere with the commitment of such precursors from the hemangioblast. These results suggested that Lhx2 expression directly affects the emerging definitive multipotential hematopoietic progenitor cell population whereas it has a limited effect on pre-hematopoietic mesoderm, limited and perhaps even a slight negative effect on the hemangioblast population.

To further study the effect of Lhx2 expression in ES cells and during ES cell differentiation and gastrulation *in vitro* we added dox to iLhx2 ES cells at different time points during differentiation and analysed day 6 EBs. Expression of Lhx2 in ES cells cultured in ES cell medium did not significantly affect their ability to self-renew, based on growth rate and viability (data not shown). Expression of Lhx2 during early ES cell commitment, e.g. from 2 days prior to initiation of differentiation (Day -2) and on day 0 and 1 of differentiation until day 6, suppressed EB formation whereas initiation Lhx2 expression after day 2 did not significantly affect the generation of EB cells (Fig. 5D). Moreover, expression of Lhx2 from day 0 and 1 lead to the formation of numerous secondary EBs when day 6 EBs were replated in clonal assays (Fig. 5E), suggesting that suppression of EB formation was most likely due to that Lhx2 expression interfered with the initial commitment step in the differentiation of ES cells. Lhx2 expression from day 0 and 1 also interfered with hematopoietic commitment as almost no hematopoietic progenitor cells were detected in day 6 EB (Fig. 5F), and expression from day 2 slightly affects hematopoietic commitment whereas expression from day 3 instead increase the frequency of definitive hematopoietic progenitor cells in day 6 EBs (Fig. 5F). Expression of the gene Brachyury has been shown to be a marker for formation of nascent mesoderm during primitive streak formation and hence a marker for initiation and progression of the gastrulation process in normal embryos as well as during ES cell differentiation *in vitro* [40], [48]. Brachyury expression during differentiation of the Ainv15 ES cells is initiated between day 2 and 3 of differentiation and reaches maximum levels at day 4 of differentiation to rapidly decline thereafter to be undetectable at day 6 of differentiation (Fig 5G). These data further support that Lhx2 expression mainly interferes with the initial step in ES cell differentiation leading to the reduction of EB cells as well as hematopoietic mesoderm, whereas it does not interfere with the gastrulation process per se.

### Lhx2 expression in intact EBs transiently induce expansion of definitive hematopoietic progenitor cells without exogenously added growth factors

**Figure 6:**
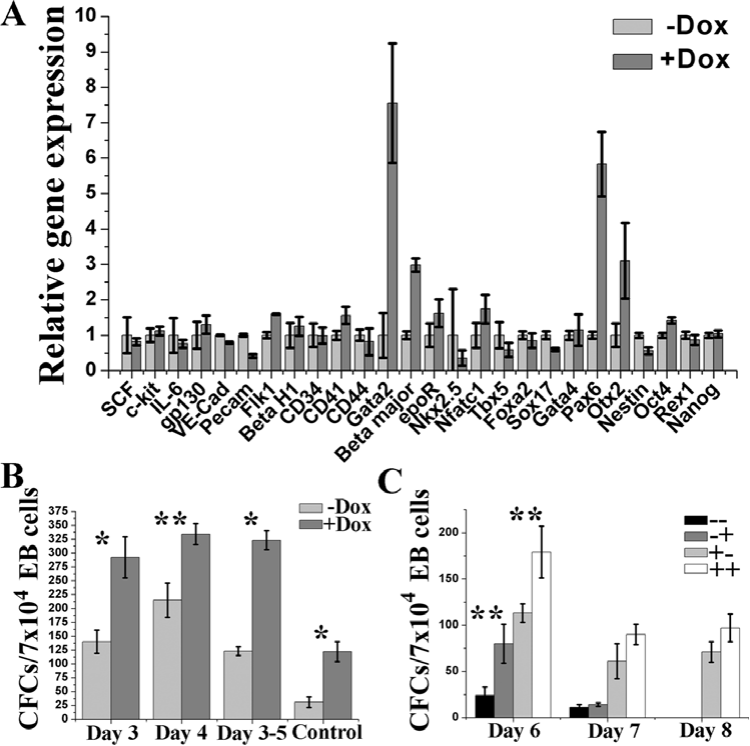
Lhx2 expression in intact EBs transiently induce expansion of definitive hematopoietic progenitor cells in the absence of exogenously added growth factors. A. Gene expression analysis by real-time PCR on day 6 EB generated from the iLhx2 ES cells comparing control EBs (−Dox) to those where Lhx2 expression was turned on at day 4 of differentiation (+Dox). B. Frequency of definitive CFCs responding to SCF/IL-6/epo in clonal assays of day 6 EBs in the presence (+Dox) or absence (−Dox) of dox, if dox was added at day 3 or 4 of differentiation, or if dox was present between day 3 and 5 of differentiation. Control is day 6 EBs generated without dox. C. Frequency of definitive CFCs responding to SCF/IL-6/epo in clonal assays of day 6, 7 and 8 EBs in the presence (−Dox) of absence (+Dox) of dox if dox was added or not at day 4 of differentiation. Hence, the following combinations were analysed: −− no dox added to the ES cells differentiation or to clonal assays of EB cells, −+ dox was added to clonal assays of day 6, 7 and 8 EB cells. +− dox was added to day 4 EB and no dox was added to clonal assays of the day 6, 7 and 8 EB cells. ++ dox was added to day 4 EBs and to the clonal assays of the day 6, 7 and 8 EB cells. Asterisks indicate where the difference in CFC between clonal assays performed in the absence or presence of dox is statistically significant, *p<0,005, **p<0,05.

The Lhx2-induced self-renewal of hematopoietic progenitor cells in clonal assays of EB cells was strictly dependent on specific and exogenously added growth factors (Fig. 3). The increase in CFCs in day 6 EBs when Lhx2 was turned on at day 3 compared to control EBs (Fig. 5F), suggested that Lhx2 expression in intact EBs support self-renewal of hematopoietic progenitor cells independently of exogenously added growth factors. To address this issue and to elucidate if Lhx2 expression in intact EBs would increase the efficiency in generating DoxHPC lines, we added dox at day 3 and 4 of differentiation and analysed day 6 EBs for progenitor cell content and gene expression pattern. Gene expression analysis of day 6 EBs generated from the iLhx2 ES cells when Lhx2 expression was turned on at day 4 showed that genes primarily expressed by definitive hematopoietic cell populations such as *GATA-2* and *β-globin* (Beta major) and early neuronal ectoderm (*Pax6* and *Otx2*) were significantly upregulated compared to control EBs, whereas genes expressed by EryP cells *(βH1* or *Beta H1*), cardiac mesoderm (*Nkx2.5*, *Nfatc1*, *Tbx1*), vascular endothelium (*VE-cadherin*, *Pecam*, *Flk-1*), endodermal cell populations (*Sox17*, *Foxa2*, *GATA4*), or various stem cell populations (*Oct4*, *Rex1*, *Nanog*, *Nestin*) were not changed (Fig. 6A). Other early hematopoietic and/or endothelial-associated genes that Lhx2 expression did not alter were *CD41*, *CD34* and *CD44*. Moreover, we did not detect any increased expression of the mediators of the c-kit and gp130 signalling pathways when Lhx2 expression was turned on in day 4 EBs (e.g. *SCF*, *c-kit*, *IL-6*, *gp130*) (Fig. 6A). The increased expression of genes associated with definitive hematopoiesis was confirmed by that the progenitor cells responding to SCF/IL6/epo had increased 5-8-fold within the intact EB when Lhx2 was expressed during this time period compared to control EBs (Fig. 6B). Furthermore, the synergistic effect on colony formation of Lhx2 expression was also observed in the clonal assays of the day 6 EBs when Lhx2 expression was induced from day 3 or 4 (Fig. 6B), indicating both self-renewal and differentiation of the distinct progenitor cell expanded by Lhx2 expression in EBs. Lhx2 expression between day 3 and 5 of differentiation lead to an equivalent expansion of progenitor cells in the EB suggesting that most of the expansion occurs between days 3-5 of differentiation (Fig. 6B). Although expression of Lhx2 in intact EBs from day 4 of differentiation increase the frequency of hematopoietic progenitors in day 6 EBs, it does not to increase the efficiency in generating DoxHPC lines from the clonal assays of day 6 EBs (65% of the picked colonies compared to 69% and 60% when dox was added only to the clonal assays, Table 1). Thus, Lhx2 expression in intact EBs leads to both self-renewal and differentiation of this distinct progenitor cell population.

During normal EB development in the absence of exogenously added growth factors the frequency of hematopoietic progenitor cells rapidly decline after day 6 of differentiation (Fig. 5A). To elucidate if Lhx2 expression in intact EBs promote expansion of hematopoietic progenitor cells to later stages when they are normally exhausted, we also analysed progenitor cell content in day 7 and 8 EBs when Lhx2 expression was turned on at day 4. This experiment revealed that Lhx2 expression maintained significant numbers of progenitor cells until day 8 EBs when they are exhausted in the control EBs (Fig. 6C). The synergistic effect of Lhx2 expression in the clonal assays of day 7 and 8 EBs was not significant as in the clonal assays of day 6 EBs, further supporting the notion that Lhx2 expression can only induced self-renewal of this distinct hematopoietic progenitor cell for a limited time in intact EBs. Thus, Lhx2 expression in intact EBs induce self-renewal the specific hematopoietic progenitor cell independently of exogenously added growth factors for a limited time (day 3–5) during EB development.

## Discussion

**Figure 7:**
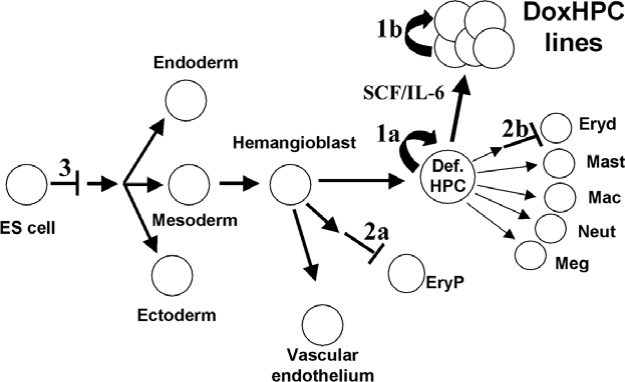
An overview of the effects of Lhx2 expression during ES cell differentiation *in vitro*. 1) Lhx2 expression and simultaneous activation of the c-kit receptor tyrosine kinase and gp130 signal transducer via IL-6 directly induce self-renewal of a distinct multipotential definitive hematopoietic progenitor cell (Def. HPC) present in the EB (1a) leading to the generation of HSC-like cell lines (DoxHPC lines) strictly dependent on Lhx2 expression and SCF/IL-6 for continuous self-renewal *in vitro* (1b). 2) Lhx2 expression inhibits proliferation of committed primitive erythroid (EryP) precursor cells (2a) and definitive erythroid (EryD) precursor cells (2b). 3) Lhx2 expression interferes with the initial step in ES cell differentiation whereas it does not interfere with the gastrulation process. Mast, mast cells. Mac, macrophages. Neut, neutrophilic granulocyte. Meg, megakaryocytes.

By using an efficient system to express Lhx2 during ES cell differentiation *in vitro* we have shown that Lhx2 expression cause three different phenotypes summarised in Figure 7: 1) self-renewal of a distinct definitive multipotential hematopoietic progenitor cell in EBs in a growth factor-specific manner, 2) complete block in proliferation and differentiation of committed EryP precursor cells and to some extent also EryD precursor cells, and 3) interference with the initial steps in ES cell differentiation *in vitro*. The effect of Lhx2 on hematopoietic progenitor cells is most likely due to a direct effect on the emerging definitive multipotential progenitor cells in the EBs and not due to an indirect effect by increasing hematopoietic commitment from pre-hematopoietic mesoderm, or by promoting proliferation of the hemangioblast cell population. The lack of effect of Lhx2 expression in clonal assays of day 3 and 4 EBs when the hemangioblast is the dominating progenitor cell population with hematopoietic potential, whereas expression in intact EBs from day 3 and 4 to day 6 of differentiation expand definitive hematopoietic progenitor cells, would suggest that Lhx2 expression does not induce self-renewal until the hemangioblast cell has matured into a progenitor cell harbouring only definitive hematopoietic potential. Moreover, non of the cell lines tested that we have generated from ES cells previously have shown any primitive hematopoietic potential [41], [47], further supporting this assumption. The frequency of the Lhx2-responsive progenitor cell within day 6 EBs in our previous work using retroviral vectors was usually less than 1 in 10^4^ EB cells [41], whereas in this study we can show that the frequency of the Lhx2-responsive progenitor cell is 1 in 1838 EB cells. This frequency corresponds to 1-2 cells per EB since each EB contain on average 2–3×10^3^ cells after 6 days of differentiation [40], whereas in our previous work the frequency corresponds to less than 1 progenitor per 3–4 EBs. This efficient and growth factor-specific induction of self-renewal of a specific multipotential progenitor cell present in EBs is unique to *Lhx2* as compared to other genes tested in the ES system, such as *Hox11*, *Hoxb4*, *Cdx4*, *Smad1*, *Stat5*, *mMixl1*, and the *BCR/ABL* oncogene [45], [49]–[57].

Signalling via the c-kit receptor and the gp130 signal transducer by IL-6 appear to be necessary and sufficient to promote Lhx2-induced self-renewal of multipotential hematopoietic progenitor cells in the ES/EB system. The growth factors that signal via gp130, IL-6, IL-11, LIF (leukemia inhibitory factor), CNTF (ciliary neurotrophic factor), OSM (oncostatin M), CT-1 (cardiotrophin-1), CLC (cardiotrophin-like cytokine) and IL-27, affect a plethora of different cell types [31], [32], and it has been shown that direct activation of gp130 does not require the specific ligand for cell type-specific signalling [58]. Interestingly, signalling via c-kit and direct activation of gp130 is also a potent regulator of human hematopoietic progenitor/stem cells further supporting the idea that modulation of these pathways profoundly influences stem cell function also in cells of human origin [59]–[61]. Moreover, c-kit receptor signalling together with gp130 signalling by IL-6 have previously been shown to be involved in the maintenance of immortalised HSC-like cell lines after transduction of HSCs with a constitutively active Notch1 receptor, or a constitutively active β-catenin that activates the canonical Wnt signalling pathway [62], [63], although the specificity or efficiency of these events are unknown. These observations suggest that c-kit signalling together with gp130 signalling are potent regulators of hematopoietic stem/progenitor cells including those of human origin, and we have shown that Lhx2 expression efficiently modulate the interplay between these receptors. To elucidate the function of Lhx2 in stem/progenitor cells we have done global gene expression analyses comparing the Lhx2^+^ DoxHPC lines to their Lhx2^−^ progeny upon dox withdrawal [47], and started to address the contribution of different signalling pathways identified in this screen. Based on the function of Lhx2 in stem/progenitor cell populations in various organ systems such as olfactory epithelium, forebrain ventricular zone, hair follicles, and the progress zone during limb development [64]–[68], elucidation of Lhx2 function would give insights into progenitor/stem cell physiology also in non-hematopoietic organs.

The generation of HSC-like cell lines in our previous work was a relatively rare event and we could not exclude the influence from secondary genetic effects due to retroviral integration, which has been shown to be a non-random event causing a proliferative advantage of the transduced clone [69]. Our inability to generate DoxHPC lines in SCF alone suggests that retroviral insertion might contribute to the generation of HSC-like cell lines in SCF alone in our previous work [41], [46]. Upregulation of endogenous IL-6 gene expression upon IL-6 withdrawal from DoxHPC lines suggests that endogenous IL-6 expression could further contribute to Lhx2 induced self-renewal in SCF alone. However, the results obtained herein exclude the influence of retroviral insertion in the generation of HSC-like cell lines and strongly suggest that induction of self-renewal of the multipotential progenitor cell is a direct and specific effect of Lhx2 expression together with c-kit and gp130 signalling.

In contrast to the strict dependence on exogenously added growth factors for Lhx2-induced self-renewal of cells in clonal assays, Lhx2 expression in intact EBs transiently induce self-renewal of these progenitor cells independent of exogenously added growth factors. This observation suggests that the EB environment can provide the growth factors required for Lhx2-induced self-renewal. SCF is expressed during ES cell differentiation *in vitro* [40], [70], [71], whereas IL-6 is not expressed during the early stages of EB development [70], and we have not seen any evidence for that Lhx2 expression in EBs upregulate IL-6 expression (Fig. 6A). We have previously shown that the HSC-like cell lines expressing Lhx2 self-renew by a cell nonautonomous mechanism which is in agreement with the cell nonautonomous hematopoietic phenotype in *Lhx2^−/−^* mice [42], [64]. Since Lhx2 is normally expressed in a mesenchymal cell population (hepatic stellate cells) in the fetal liver and these cells are important for the differentiation, organization and expansion of all cell types in the liver including those of the hematopoietic niche [64], [72], it would suggest that Lhx2 regulates genes in mesenchymal cells encoding mediators involved in cell-to-cell interactions. Lhx2 expression in mesenchymal cells in addition to expression in hematopoietic cells might therefore enhance the effect on hematopoietic cells in intact EBs and the transient nature of the expansion (day 3–5 of EB development) suggests that such interactions are only possible during early EB development. Elucidation of the molecular basis for Lhx2-induced self-renewal might therefore give insights into both cell autonomous and cell nonautonomous mechanisms regulating normal HSCs function.

Another obvious effect of Lhx2 expression on hematopoietic precursors was the complete block in proliferation of committed EryP precursor cells and to some extent also of EryD precursor cells. This observation could explain the large fluctuation in erythropoiesis *in vivo* seen in stem cell-deficient mice engrafted with adult HSC-like cell lines expressing Lhx2, whereas generation of myeloid cells appeared to be relatively unaffected [46]. Since the level of Lhx2 expression is critical for the inhibition of erythroid development, these fluctuations could simply reflect fluctuations in the level of Lhx2 expression in erythroid precursors, and hence when Lhx2 expression is below a critical threshold the erythroid precursor can escape the inhibition. We also noticed a significantly decreased mean corpuscular hemoglobin concentration (MCHC) in erythrocytes originating from the HSC-like cell lines in the engrafted animals [73], suggesting that Lhx2 expression could interfere with globin expression. The *Lhx2* gene encodes a LIM-domain-containing transcription factor and transcriptional regulators containing LIM-domains (Lmo2) and LIM-domain-interacting domains (Ldb1) are known to be critically involved in erythroid differentiation [74]–[76]. Since Lhx2 is not normally expressed in hematopoietic cells, the LIM-domain of the Lhx2 protein might physically interfere with these protein interactions and depending on the amount of Lhx2 protein in the cell cause dominant negative effects leading to the block in erythroid development and/or altered globin expression.

It has been suggested that expression of the *Lhx2* gene is repressed in ES cells by the Polycomb complex [77], [78], implying that Lhx2 would promote differentiation of ES cells. However, our data reveal that Lhx2 expression does not have any obvious effects on the self-renewal of ES cells but it does interfere with the early steps in ES cell differentiation leading to reduced number of EB cells and suppression of hematopoietic mesoderm. These effects are only seen if Lhx2 is turned very early during ES cell differentiation (primarily from day 0 and 1) whereas expression after day 2 has subtle effects on the differentiation. Since our differentiation protocol is prone towards mesodermal commitment and is inefficient in promoting ectodermal and endodermal commitment, our results can be interpreted in at least two different ways. Firstly, Lhx2 interferes with conversion of primitive ectoderm to more differentiated tissues. Secondly, Lhx2 expression promotes differentiation towards a lineage that is not supported by our differentiation protocol. One of the first cell types expressing Lhx2 in the developing embryo is the neuronal ectoderm of the prospective forebrain prior to E9 (unpublished observation) and gene expression analysis of the day 6 EBs when Lhx2 was turned on at day 4 revealed a significant upregulation of the forebrain markers Pax6 and Otx2. These results suggest that Lhx2 might promote neuronal differentiation which is not supported by our differentiation protocol and neuronal commitment might be more efficient if Lhx2 is turned very early during ES cell differentiation and hence cause the reduced formation of EB cell and suppression of mesodermal commitment. Which of these alternatives that are correct are presently under investigation. Collectively these results show that the effects of Lhx2 expression have striking lineage specificity and that the level of expression is important for these effects. This work provides the first attempt to understand the wide array of functions that Lhx2 have in different organs during embryonic development such as the hematopoietic system, the liver, forebrain and neuronal retina.

## Materials and Methods

### Generation of ES cells with inducible gene expression

The Ainv15 ES cell line was maintained on irradiated mouse embryonic feeder (MEF) cells in Dulbeccós modified Eagle medium (DMEM) (Gibco-BRL, United Kingdom) supplemented with 15% fetal calf serum (FCS) (Boehringer, Germany), 1.5×10^−4^ M monothioglycerol (MTG) (Sigma, Germany) and LIF (Chemicon, Ca, USA). Green fluorescent protein (GFP) cDNA, Lhx2 cDNA or Lhx2 cDNA linked to a GFP cDNA preceded by an internal ribosomal entry site (Lhx2-GFP), was inserted into the plox vector and transfected into the Ainv15 ES cells together with Cre recombinase cDNA as previously described [45]. The plox vector will be inserted into the loxp site down stream of the tet-responsive element (TRE) adjacent to the *Hypoxanthine-phosphoribosyltransferase* (*HPRT*) gene by recombination between the chromosomal and plasmid loxp sites (Fig. 1A). Reconstitution of a functional *Neo* gene by the promoter-ATG sequence (PGK-ATG) allows for selection of successful integration events (Fig. 1A), and hence transfected ES cells were subsequently cultured in 200 µg/ml G418 (Gibco-BRL). Clones of G418^R^ ES cells transfected with the respective gene construct were isolated, pooled and expanded. The resulting cell lines are referred to as iGFP, iLhx2 and iLhx2-GFP ES cell lines, respectively. Induction of gene expression was carried out by adding dox to a final concentration of 2 µg/ml of dox if not stated otherwise. Induction of Lhx2 expression was done using the iLhx2 ES cell line if not stated otherwise.

### Differentiation of ES cell *in vitro*

The iGFP, iLhx2 and iLhx2-GFP ES cell lines used for *in vitro* differentiation were made feeder-independent in serum-free medium as previously described [79], [80]. Briefly, ES cells cultured on MEF cells were treated with Acutase (Chemicon International, Cat. No. SF006), transferred to gelatinized culture flasks in ESGRO Complete Clonal grade medium (Chemicon International, Cat. No. SF001) and passaged in this medium until no MEFs were present and were subsequently maintained in this medium by 1∶4 to 1∶5 splits. For differentiation, ES cells were treated with Acutase , washed and transferred at various densities (10^3^−8×10^3^ cells/ml) into Iscovés modified Dulbeccós media (IMDM) (Gibco-BRL) supplemented with 15% FCS (Integro Inc., The Netherlands), 4.5×10^−4^ M MTG, 5% Protein Free Hybridoma Medium II (PFHMII, GIBCO) and 25 µg/ml ascorbic acid (Sigma). EBs were collected after different days of differentiation, resuspended in Acutase and incubated for three minutes. Two ml of FCS was added and the cells were gently passaged through a syringe with a 20-gauge needle. Ten ml of IMDM supplemented with MTG was added; the cells were spun down and resuspended in fresh IMDM medium supplemented with MTG and 5% FCS.

### Clonal progenitor cell assays of EB cells

The clonal assays were carried out in IMDM containing 1% methylcellulose (Fluka, Switzerland) and supplemented with L-glutamine, 300 µg/ml iron-saturated transferrin (Boehringer), 5% PFHMII, 10% plasma-derived serum (Antech Inc., Tx, USA), with or without doxycyclin (dox) (Sigma) and with the indicated growth factors. Growth factors used were: mouse SCF (R&DSystems) at 100 ng/ml, human IL-6 (R&DSystems) at 10 ng/ml, mouse thrombopoietin (tpo) (R&DSystems) at 20 ng/ml, mouse Flt3L (R&DSystems) at 20 ng/ml, IL-3 at 1% of conditioned media from a cell line transfected with mouse IL-3 cDNA [81] and human erythropoietin (epo) (Eprex Janssen-Cilag, Sweden) at 4 IU/ml. EB cells were plated in triplicates in a final volume of 1.25 ml in 35-mm Petri dishes (Falcon 1008) at 7×10^4^ to 2×10^5^ cells/dish. The frequency of EryP precursor cells was determined by scoring the number of EryP colonies after 5 days of incubation and the frequency of definitive colony-forming cells (CFCs) was determined by scoring the number of definitive hematopoietic colonies after 9–10 days of incubation.

### Generation and maintenance of DoxHPC lines

Clonal assays containing either SCF/IL-6/epo/dox or SCF/epo/dox were performed on day 6 EBs generated from the iLhx2 and iLhx2-GFP ES cell lines. Individual colonies were randomly picked from the clonal assays after 9–10 days of incubation, transferred to 96-well plates and expanded in IMDM supplemented with 5% FCS, 1.5×10^−4^ M MTG, and SCF/IL-6/dox or SCF/dox. The individual colonies were cultured for at least 3 weeks after which the cells were analysed for cell morphology by May-Grünwald Giemsa staining of cytospun cells, and at this stage the cultures contained cells either with mast cell morphology or blast cell-like morphology (ref. 47 and data not shown). All cultures containing cells with blast cell-like morphology at this time point generated stable cell lines and are referred to as dox-dependent hematopoietic progenitor cell (DoxHPC) lines. The DoxHPC lines were subsequently maintained in this media at cell densities between 5×10^5^ and 2×10^6^ cells/ml as previously described for other Lhx2-induced HSC-like cell lines (HPC and BM-HPC lines) [42], [46]. The cultures containing cells with mast cell morphology remained as such despite maintained Lhx2 expression. The DoxHPC lines down regulated Lhx2 expression by >95% within 24 hrs after dox withdrawal and all DoxHPC lines tested thus far are multipotential as they differentiate into various myeloid cells (megakaryocyte, macrophages, neutrophilic granulocytes, mast cells) upon dox withdrawal [47]. No cell line could be established from EBs generated from the control iGFP ES cell line in the presence of dox, or from EBs generated from the iLhx2 ES cell line in the absence of dox, as such cells differentiated into mast cells under these culture conditions.

### Gene expression analysis

Total RNA was extracted from cell pellets with RNeasy Plus Mini Kit (Qiagen). cDNA was synthesized using the First-strand cDNA synthesis Kit (Amersham Biosciences). Real-time PCR reactions were carried out in triplicates using SYBR green PCR master mix (Applied Biosystems Ca. US) and PCR products were detected with an ABI prism 7000 instrument (Applied Biosystems). The expression levels of the genes tested were normalized to the expression levels of house keeping gene *Gapdh* (Glyceraldehyde-3-phosphate dehydrogenase) and confirmed with one additional house keeping gene *Tbp* (TATA box binding protein). The following primers were used: Gapdh forward primer (F) CGTGTTCCTACCCCCAATGT and reverse primer (R) TGTCATCATACTTGGCAGGTTTCT, Tbp F GAATTGTACCGCAGCTTCAAAA and R AGTGCAATGGTCTTTAGGTCAAGTT, CD34 F CTTGGGCACCACTGGTTATTTC and R GGTCTTCACCCAGCCTTTCTC, CD41 F GCTCCGGCTCACAGCTACTG and R ATCATTGGCTGCTTCAATCTTCA, Gata2 F GGCACGGGCCACTACCT and R TCGTCTGACAATTTGCACAACAG, βH1 F AGGCAGCTATCACAAGCATCTG and R AACTTGTCAAAGAATCTCTGAGTCCAT, β major F GTGAGCTCCACTGTGACAAGCT and R GGTGGCCCAGCACAATCACGATC, Flk1 F ACTGCAGTGATTGCCATGTTCT and R TCATTGGCCCGCTTAACG, SCF F GAAAACGCACCGAAGAATATAAAAG and R TCTAGTTTCTGGCCTCTTCGGA, c-kit F CACTCGCACGGGCACAT and R AAGTTTGGCAGGATCTCTAACAAAC, CD44 F TCCGAATTAGCTGGACACTCAA and R TCTCCTCATAGGACCAGAAGTTGTG, IL-6 F ACAAGTCGGAGGCTTAATTACACAT and R AATCAGAATTGCCATTGCACAA, gp130 F TGCTGGGCGTCTTGTTCTG and R ATATGACTCTTGGAAGGATCAGGAA, epoR F GGATGGACTTCAACTACAGCTTCTC and R CCTGGTGCAGGCTACATGACT, Otx2 F GAGCTCAGTCGCCACCTCTACT and R CCGCATTGGACGTTAGAAAAG, Pax6 F CCACCCATGCCCAGCTT and R AACTGACACTCCAGGTGAAATGAG, Oct4 F TGAGCC>GTCTTTCCACCA and R TACCTCCCTTGCCTTGGC, GATA4 F CACCCCAATCTCGTAGATATGTTTG and R GGTAGTGTCCCGTCCCATCTC, VE-Cad F AGCGCAGCATCGGGTACT and R GTTATAGATGTTTCCCTGCTTGGTTAT, Pecam F CTGCAGGCATCGGCAAA and R GCATTTCGCACACCTGGAT, Nkx2.5 F CCAAGTGCTCTCCTGCTTTCC and R GCCATCCGTCTCGGCTTT, Nfatc1 F CCATACGAGCTTCGGATCGA and R AGTAACCGTGTAGCTGCACAATG, Tbx5 F CGTTTGGACACATTATCCTGAACT and R TGAACCGAACCCATTATTTTCG, Nestin F CTCTTCCCCCTTGCCTAATACC and R TTTAGGATAGGGAGCCTCAGACAT, Foxa2 F GGCCAGCGAGTTAAAGTATGCT and R CTCGGGCTCCGCGTAGTAG, Sox17 F CCCAACACTCCTCCCAAAGTATC and R TTCCCTGTCTTGGTTGATTTCTC, Rex1 F TCACTGTGCTGCCTCCAAGT and R GGGCACTGATCCGCAAAC, Nanog F AAACCAGTGGTTGAAGACTAGCAA and R TGCAATGGATGCTGGGATACT.

Reverse transcriptase (RT) PCR was performed using Taq polymerase (Amplicon, Denmark) on cDNA prepared from feeder-independent iLhx2 ES cells cultured without dox (0 hrs) and in 2 µg/ml of dox for 6, 12, 18 and 24 hours. The samples were loaded on the 1% Agarose gel and the PCR primers used were Lhx2 F AAAAGACAAAGCGCATGCGGC and R CAGGCACAGAAGTTAAGACTG, Hprt F CACAGGACTAGAACACCTGC and R GCTGGTGAAAAGGACCTCT.

GFP expression analysis was carried in a FACSCalibur™ (Becton Dickinson, Ca USA) using CellQuestPro measuring fluorescence in the FL1 channel on the feeder-independent iLhx2-GFP ES cells cultured as described previously in the presence or absence of 2 µg/ml of dox for 2 days.

### Statistical analysis

Data are presented as mean±standard deviation (SD). p values were calculated using Student's t test and p values <0,05 were considered as statistically significant.
